# MGMT Methylation and Differential Survival Impact by Sex in Glioblastoma

**DOI:** 10.3390/cancers16071374

**Published:** 2024-03-31

**Authors:** Addison E. Barnett, Ahmad Ozair, Anas S. Bamashmos, Hong Li, David S. Bosler, Gabrielle Yeaney, Assad Ali, David M. Peereboom, Justin D. Lathia, Manmeet S. Ahluwalia

**Affiliations:** 1 Rose Ella Burkhardt Brain Tumor & Neuro-Oncology Center, Cleveland Clinic, Cleveland, OH 44106, USA; abarnett21618@med.lecom.edu (A.E.B.); anas.saeedbamashmos@nyulangone.org (A.S.B.); alias@mlhs.org (A.A.); peerebd@ccf.org (D.M.P.); lathiaj@ccf.org (J.D.L.); 2 Miami Cancer Institute, Baptist Health South Florida, Miami, FL 33176, USA; aozair1@jh.edu; 3 Bloomberg School of Public Health, Johns Hopkins University, Baltimore, MD 21218, USA; 4 NYU Langone Health, New York, NY 10016, USA; 5 Department of Quantitative Health Sciences, Cleveland Clinic, Cleveland, OH 44106, USA; lih@ccf.org; 6 Robert J. Tomisch Pathology & Laboratory Medicine Institute, Cleveland Clinic, Cleveland, OH 44106, USA; boslerd@ccf.org (D.S.B.); yeaneyg@ccf.org (G.Y.); 7 Lerner Research Institute, Cleveland Clinic, Cleveland, OH 44106, USA; 8 Herbert Wertheim College of Medicine, Florida International University, Miami, FL 33199, USA

**Keywords:** glioblastoma, sexual dimorphism, O6-methylguanine-DNA-methyltransferase, MGMT, cysteine–phosphate–guanine, CpG

## Abstract

**Simple Summary:**

Glioblastoma is a highly aggressive and lethal brain tumor that has seen marginal improvement in patient outcomes despite decades of concerted efforts. This study investigated the impact of tumor molecular features, sex, and their interaction on the survival of patients with newly diagnosed glioblastoma. Our findings show that females are more often found to have silencing of the MGMT promoter, but that they also receive a greater survival benefit, which is more clinically and statistically significant, associated with MGMT promoter silencing that is not reflected in males. These findings may significantly impact both our understanding as well as the clinical management of the disease. Rather than the established practice of using temozolomide to treat MGMT promoter methylated patients as a whole, our findings suggest that females accrue a disproportionate survival benefit compared to males who, regardless of methylation status, may experience better survival outcomes from alternative treatment options.

**Abstract:**

**Introduction**: Sex differences in glioblastoma (GBM) have been observed in incidence, genetic and epigenetic alterations, and immune response. These differences have extended to the methylation of the MGMT promoter, which critically impacts temozolomide resistance. However, the association between sex, MGMT methylation, and survival is poorly understood, which this study sought to evaluate. **Methods**: A retrospective cohort study was conducted and reported following STROBE guidelines, based on adults with newly diagnosed GBM who received their first surgical intervention at Cleveland Clinic (Ohio, USA) between 2012 and 2018. Kaplan–Meier and multivariable Cox proportional hazards models were used to analyze the association between sex and MGMT promoter methylation status on overall survival (OS). MGMT was defined as methylated if the mean of CpG 1-5 ≥ 12. Propensity score matching was performed on a subset of patients to evaluate the effect of individual CpG site methylation. **Results**: A total of 464 patients had documented MGMT methylation status with a mean age of 63.4 (range 19–93) years. A total of 170 (36.6%) were female, and 133 (28.7%) received gross total resection as a first intervention. A total of 42.5% were MGMT methylated, with females more often having MGMT methylation than males (52.1% vs. 37.4%, *p* = 0.004). In univariable analysis, OS was significantly longer for MGMT promoter methylated than un-methylated groups for females (2 yr: 36.8% vs. 11.1%; median: 18.7 vs. 9.5 months; *p* = 0.001) but not for males (2 yr: 24.3% vs. 12.2%; median: 12.4 vs. 11.3 months; *p* = 0.22, *p* for MGMT–sex interaction = 0.02). In multivariable analysis, MGMT un-methylated versus methylated promoter females (2.07; 95% CI, 1.45–2.95; *p* < 0.0001) and males (1.51; 95% CI, 1.14–2.00; *p* = 0.004) had worse OS. Within the MGMT promoter methylated group, males had significantly worse OS than females (1.42; 95% CI: 1.01–1.99; *p* = 0.04). Amongst patients with data on MGMT CpG promoter site methylation values (*n* = 304), the median (IQR) of CpG mean methylation was 3.0% (2.0, 30.5). Females had greater mean CpG methylation than males (11.0 vs. 3.0, *p* < 0.002) and higher per-site CpG methylation with a significant difference at CPG 1, 2, and 4 (*p* < 0.008). After propensity score matching, females maintained a significant survival benefit (18.7 vs. 10.0 months, *p* = 0.004) compared to males (13.0 vs. 13.6 months, *p* = 0.76), and the pattern of difference was significant (P for CpG–sex interaction = 0.03). **Conclusions**: In this study, females had higher mean and individual CpG site methylation and received a greater PFS and OS benefit by MGMT methylation that was not seen in males despite equal degrees of CpG methylation.

## 1. Introduction

Glioblastoma (GBM) is the most common primary malignant central nervous system tumor among adults [1]. The prognosis of GBM is dismal, with a median survival of 15–18 months and a 5.6% 5-year survival rate [1,2]. The current standard of care for GBM includes maximal surgical resection followed by concomitant temozolomide chemo-radiation and adjuvant temozolomide. This combination of chemo-radiotherapy improved overall survival outcomes from 12.1 to 14.6 months [2]. The O^6^-methylguanine-DNA methyltransferase (MGMT) gene encodes the DNA repair enzyme that removes O^6^-methylguanine base adducts, thereby protecting against G:C → A:T mutations, and repairs damage induced by alkylating chemotherapeutic agents [3,4,5,6,7,8]. Within the DNA are high-concentration regions of cysteine–phosphate–guanine (CpG) dinucleotides. A region of greater than 200 base pairs and a CpG concentration of greater than 50% is considered a CpG island, comprised of individual CpG sites, which occur in high frequency in promoter regions but are otherwise underrepresented in the DNA. This underrepresentation is attributed to active CpG suppression as they are prone to methylation. Methylated cysteine can spontaneously convert into thymine through deamination thus resulting in mutations. Sufficient epigenetic methylation of the MGMT promoter region inhibits the enzyme’s ability to repair aberrant DNA damage as well as chemotherapy-induced cytotoxicity. Thus, higher degrees of promoter methylation predict higher chemo-sensitivity by low MGMT repair activity, and vice versa. Patients with MGMT promoter methylation have a median overall survival of 21.7 months compared to 12.7 months in the un-methylated population [3].

Recent studies have begun to identify and characterize the sexual dimorphism in GBM and MGMT, which reveals that males and females with GBM have different outcomes [1,4,9,10,11,12,13,14,15,16,17,18,19,20,21]. Primary GBM is 1.58 times more common among male than female patients and females have better outcomes when adjusted for clinical variables [1,20]. In contrast, females have higher incidence rates of secondary GBM and chemotherapy-related myelotoxicity [14,15].

The objective of this study was to investigate sex-associated rates of dichotomous MGMT promoter methylation (methylated vs. un-methylated) as well as the degree of MGMT CpG promoter site methylation and their associated outcomes. We investigated whether overall survival (OS) and progression-free survival (PFS) exhibit a sex-associated relationship with MGMT promoter methylation. These findings may influence the interpretation of prognostic factors as they relate to a patient’s sex, as well as clinical practice in the use of temozolomide.

## 2. Materials and Methods

### 2.1. Design, Ethics, Reporting, Patient Selection, and Data Collection

**Figure 1:**
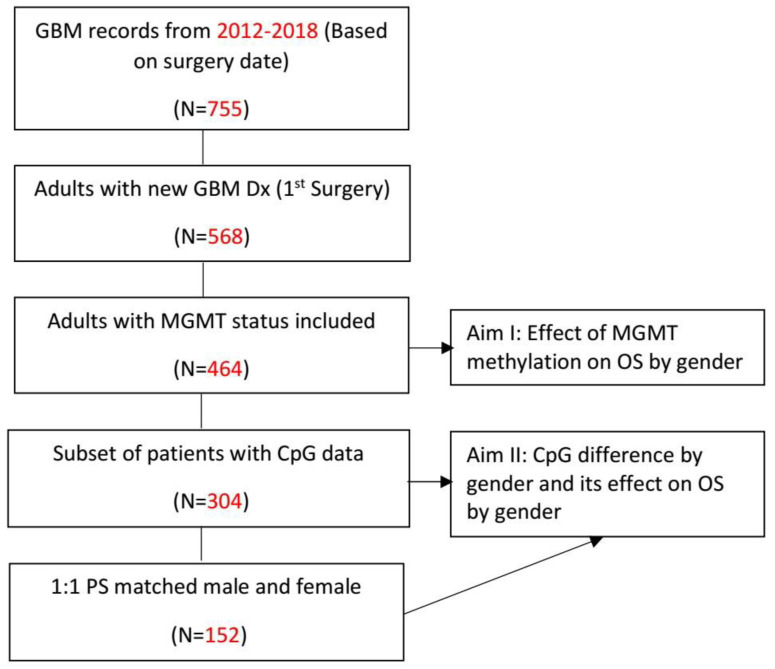
Flowchart of study inclusion (*n* = 464 overall) and selection of subset of patients.

A retrospective cohort study was conducted and reported following “strengthening the reporting of observational studies in epidemiology” (STROBE) guidelines. The work was approved by the Institutional Review Board of Cleveland Clinic, Ohio (reference number 09-911) before commencement. Adult patients with newly diagnosed GBM were evaluated for inclusion, with primary analyses conducted on those with available data on MGMT methylation status. Inclusion of patients by available data and assessment criteria is illustrated in Figure 1.

### 2.2. MGMT and CpG Methylation Analysis

**Figure 2:**
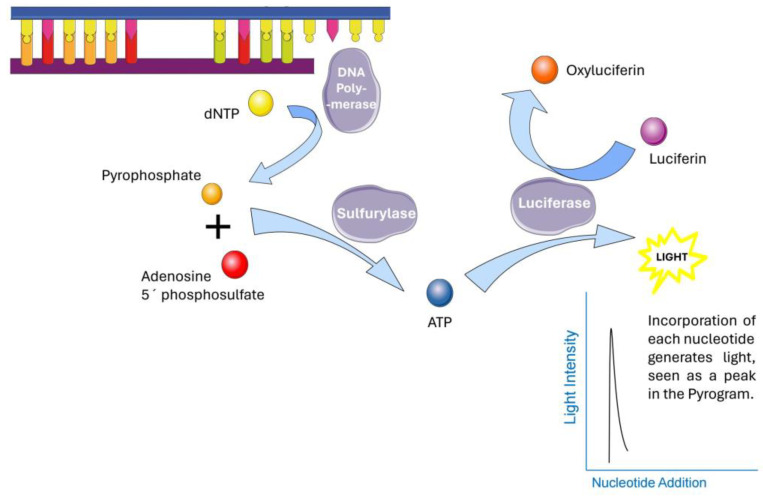
Fundamentals of the pyrosequencing reaction used to determine CpG methylation, and the MGMT promoter methylation status. (Original figure).

**Figure 3:**
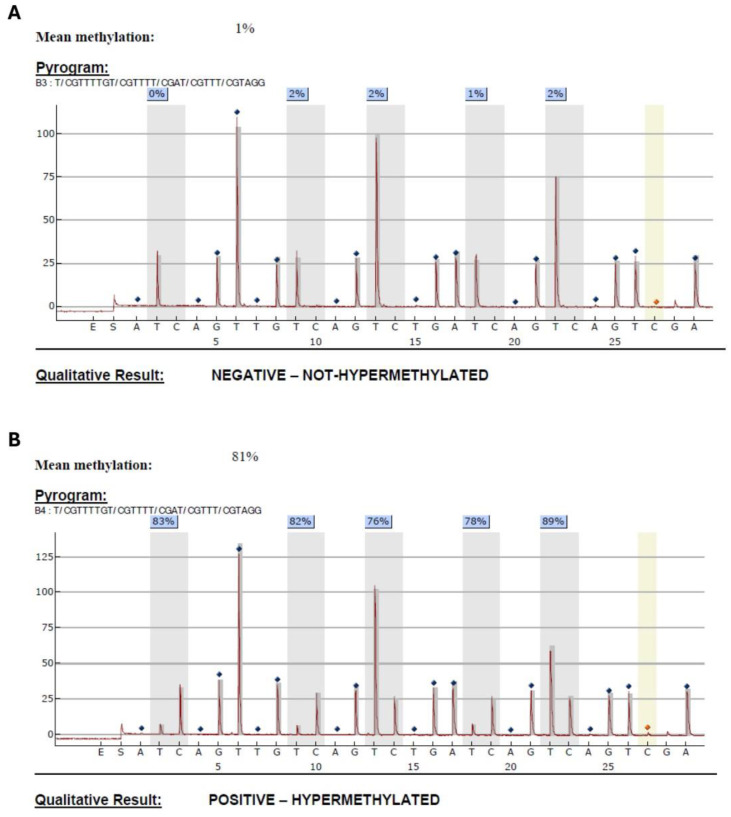
Readouts of the actual pyrosequencing platform, showing both MGMT un-methylated (**A**) and MGMT methylated examples (**B**). The five loci that are interrogated are grey-colored and methylation % at each location (i.e., how much of the cytosine has been converted to thymine), as well as the mean methylation % across these five sites at the top is also visualized. During the pyrosequencing procedure, the different bases (dNTPs) are added on a rotating basis (in the order listed along the bottom), and the base is indicated to be present (next in the sequence) only if a peak is present. The sequence is read from left to right using the peaks and peak heights, with higher peaks corresponding to duplicated bases in the sequence. The five potentially methylated sites are Ts if un-methylated (having been converted by the bisulfite reaction) and they are Cs if methylated (having been prevented from bisulfite conversion by the methyl group). The method uses a ratio of peak heights of the “C” and “T” signals at each of the sites to calculate percent methylation, and then an average across the five sites is calculated for the final methylation %.

Within the MGMT gene are over 200 identified CpG sites. Among these, several individual CpG sites as well as grouped CpG site means have been analyzed and found to be most prognostic of their suppression of MGMT transcription. In this work, MGMT promoter methylation was determined by a clinically validated test using bisulfite conversion followed by PCR and pyrosequencing of CpG sites 74–78 within nucleotides 28–52 [22,23,24]. Figure 2 provides a simplified overview of the detection reaction and Figure 3 provides an actual readout of the pyrosequencing with detailed description. Amongst the 565 patients with available dichotomous MGMT methylation data, 464 had documented MGMT promoter methylation status. A total of 304 patients had available CpG methylation site data (CpG1-CpG5). The mean percentage of methylation was used to determine methylation status [5,22,23,24,25,26].

### 2.3. Statistical Analysis

Categorical clinical and pathologic variables were summarized as frequency counts and percentages. Continuous variables were summarized as means and standard deviation and compared between gender and MGMT status using *t* test and chi-square test. F Primary analysis focused on MGMT promotor methylation effect on clinical outcomes of progression-free survival (PFS) and overall survival (OS) in males and females. All analyses were performed using SAS version 9.4. Two-sided *p*-values are presented, *p* < 0.05 was considered statistically significant.

Time from surgery to death or last contact was calculated for both PFS and OS analysis. Factors potentially associated with PFS and OS were identified using Kaplan–Meier estimation and univariable Cox proportional hazard model. Two variables (KPS before surgery and EGFR amplification) had a lot missing (≥15%), since they were similar between both gender and MGMT status groups, they were not included in clinical outcome analyses. One variable (IDH mutation) had 15% missing since it was similar between gender groups and less than 5% patients (*n* = 22) with the mutation, and 16/22 with MGMT methylation, it was also not included in clinical outcome analyses. Pattern of MGMT impact by sex on OS was determined using an interaction term in Cox hazard model. Because of significant sex–MGMT interaction, sex (White/other race) and MGMT methylation (Yes/No) were combined into categories of female/un-methylated, female/methylated, male/un-methylated, and male/methylated in analyses. Factors that were potentially associated with OS (*p* < 0.05) were included in a multivariable Cox proportional hazards model with contrast for multiple comparisons of sex and MGMT groups.

Each CpG parameter and mean of CpG1-CpG5 were compared between males and females using Wilcoxon rank test and mean CpG ≥ 12 was compared using chi-square test. A propensity score matching in logistic regression model was performed to identify males and females with same CpG mean value, and 1:1 CpG exact match in Greedy method was used. A total of 152 patients fit the criteria for direct propensity score matching analysis. Pattern of MGMT impact by sex on OS was examined in matched sample using same method mentioned above.

## 3. Results

A total of 568 patients who underwent the first surgical intervention at Cleveland Clinic Brain Tumor Center between 2012 and 2018 were evaluated for inclusion. A total of 464 of 582 patients (79.9%) who had documented MGMT promoter methylation status were included in the primary analyses. The mean age in these 464 patients was 63.4 (19–93) years, 170 (36.6%) were female, and 133 (28.7%) received gross total resection. The overall rate of MGMT promoter methylated patients was 42.5%, although females more often had promoter methylation compared to males (52.1% vs. 37.4%, *p* = 0.004). Baseline patient characteristics, overall and stratified by sex, are summarized in Table 1. Characteristics stratified by MGMT methylation status are summarized in Table 2.

### 3.1. Sex, MGMT Methylation Status and Clinical Outcomes

**Figure 4:**
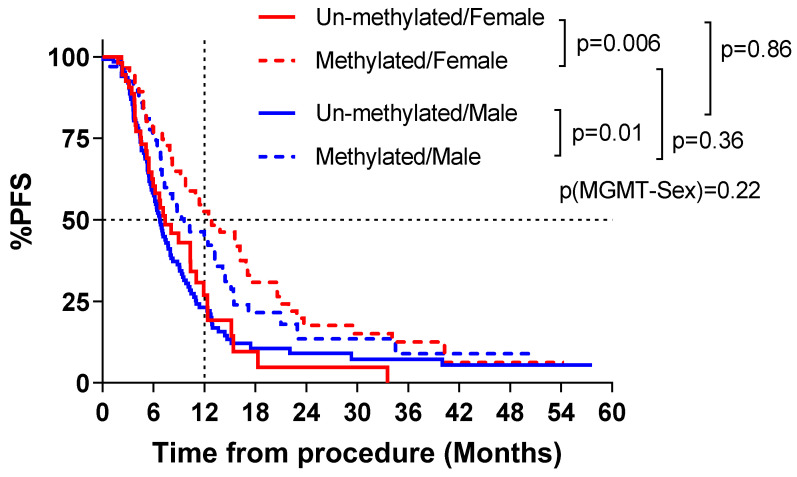
Kaplan–Meier survival estimate of progression-free survival (PFS) by sex and methylation status.

**Figure 5:**
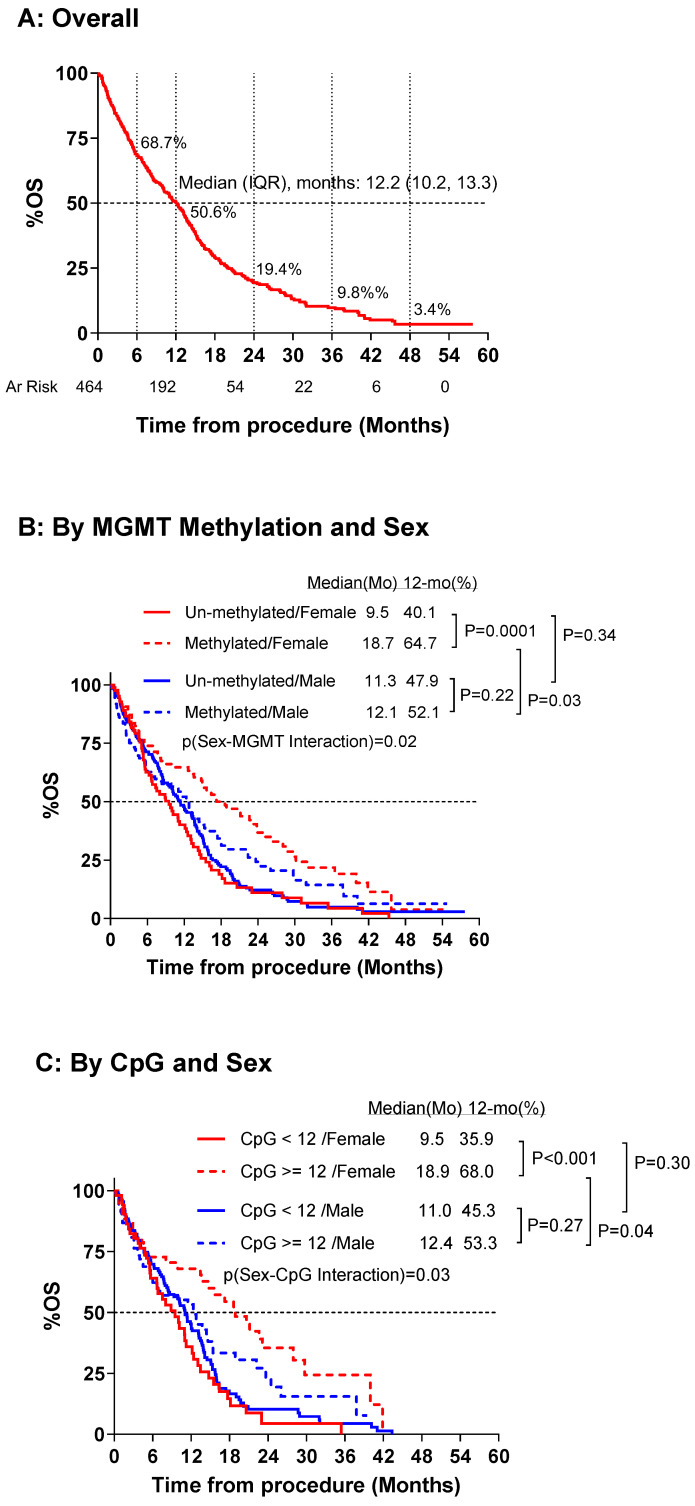
Kaplan–Meier survival estimate of overall survival (OS) by sex and methylation status.

In univariable analyses, PFS was significantly longer for MGMT promoter methylated than un-methylated groups for both males (1 yr: 44.4% vs. 23.2%; median: 9.6 vs. 6.8 months; *p* = 0.01) and females (1 yr: 52.6% vs. 26.9%; median: 12.8 vs. 7.4 months; *p* = 0.006). However, the pattern of difference was not statistically significant (P for MGMT–sex interaction = 0.22), females tended to have a greater PFS difference by promoter methylation status compared to males (1 yr difference: 25.7% vs. 21.1%; median time difference: 5.4 vs. 2.8 months), Figure 4. Further analyses focused on OS. Overall survival was significantly longer for MGMT promoter methylated than un-methylated groups for females (2 yr: 36.8% vs. 11.1%; median: 18.7 vs. 9.5 months; *p* = 0.001) but not for males (2 yr: 24.3% vs. 12.2%; median: 12.4 vs. 11.3 months; *p* = 0.22). MGMT promoter methylated females had significantly longer OS compared to methylated males (2 yr: 36.8% vs. 24.3%; median 18.7 vs. 12.4 months, *p* = 0.03). Additionally, females had a significant OS difference by promoter methylation status compared to males (2 yr difference: 25.7% vs. 12.1%; median difference: 9.2 vs. 1.1 months; *p* = 0.02), Figure 5.

In multivariate analysis, adjusted for age and surgery type, MGMT promoter un-methylated versus methylated females (1.89; 95% CI, 1.21–2.95; *p* = 0.002) and males (1.62; 95% CI, 1.13–2.32; *p* = 0.005) had a worse PFS, Table 3. Similarly, after adjustment, MGMT promoter un-methylated versus methylated females (2.07; 95% CI, 1.45–2.95; *p* < 0.0001) and males (1.51; 95% CI, 1.14–2.00; *p* = 0.004) had worse OS. Within the MGMT promoter methylated group, sex difference was not significant on PFS (1.23; 95% CI: 0.81–1.87; *p* = 0.39), however, males had significantly worse OS than females (1.42; 95% CI: 1.01–1.99; *p* = 0.04).

### 3.2. Impact of Site-Specific CpG Methylation Status on OS

**Figure 6:**
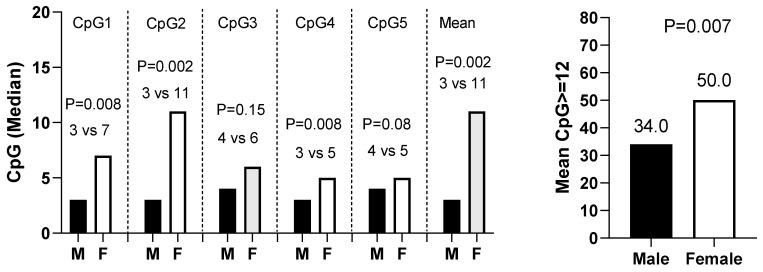
Examining association between sex and CpG status (*n* = 304).

A total of 304 patients had data available on individual site-specific CpG methylation status. The mean age overall was 63.8, with 34.2% female. A total of 39.5% of patients were MGMT methylated and the median (IQR) of CpG mean methylation levels was 3.0% (2.0, 30.5). More females were MGMT methylated than males (50.0 vs. 34.0%) and had greater mean CpG methylation than males (11.0 vs. 3.0), *p* < 0.002, Figure 6. Females had increased methylation at each CpG site, with a significant difference at CPG 1, 2, and 4 (*p* < 0.008). Before matching (*n* = 304), MGMT methylated versus un-methylated females had significantly increased median and 1-year survival (18.9 vs. 9.5 months, 68.0 vs. 35.9%, *p* = 0.0004) compared to males (12.4 vs. 11.0 months, 53.3 vs. 45.3%, *p* = 0.27), *p* for CpG–sex interaction = 0.03 (Figure 5C and Table 4). After propensity score matching (*n* = 76 each, total *n* = 152), females maintained a significant survival benefit (18.7 vs. 10.0 months, 78.4 vs. 37.4%, *p* = 0.004) compared to males (13.0 vs. 13.6 months, 56.0 vs. 56.7%, *p* = 0.76, *p* CPG-sex interaction = 0.048, Table 4).

## 4. Discussion

The role of sex in differentially impacting the survival benefit of MGMT methylation status in gliomas is being increasingly recognized [1,4,9,10,11,12,13,14,15,16,17,18,19,20,21]. The findings of this study highlight a profound sex difference in GBM patient outcomes in a sex-specific manner, whereby MGMT methylation is not prognostic in males and can markedly separate survival in female patients. We also observed sex differences in the percentage of GBM patients with MGMT methylation, similar to a 2008 study of 371 primary GBM patients where Zawlik et al. first reported that females more often have MGMT promoter methylation compared to males (53% vs. 39%; *p* = 0.0106), similar to our findings [4]. A 2016 study by Schiffgens et al. investigated the relative benefit of MGMT promoter methylation by sex where MGMT promoter methylation was significantly associated with longer survival independent of sex (*p* = 0.009) [10]. However, when dichotomized by sex, promoter methylation was only significantly associated with longer survival in females (*p* = 0.003) and not in males (*p* = 0.603). A secondary analysis of GBM patients who underwent surgery followed by chemo-radiotherapy showed that MGMT promoter methylation was significantly associated with longer survival in the entire cohort (*p* = 0.003) and in females (*p* = 0.008) but again not in males (*p* = 0.252). A 2018 study by Franceschi et al. reported prospective MGMT promoter methylation data in 140 GBM patients. They demonstrated a greater prevalence of MGMT promoter methylation in females compared to males (56% vs. 43%) [11]. On univariate and multivariate analysis of sex and MGMT promoter methylation status, a significant association was identified. Females with MGMT promoter methylation had a significant survival benefit compared to methylated males, and 1-year OS for methylated females was significantly greater compared to methylated males (78.1% vs. 66.7%; *p* = 0.028). Smits et al. further emphasized this trend in their 2021 reanalysis of two prior research cohorts [17]. Beyond confirming previous findings of sex-associated rates of methylation status and survival outcomes, they highlighted the female MGMT methylated subgroup as an outlier with regard to survival benefit. A more comprehensive profile on the nature of sexual dimorphism and immune function in male and female GBM treated with immunotherapy was described by Shireman et al. in 2022. In addition to improved survival outcomes in females compared to males, their research showed that “sexually dimorphic genes tend to enrich for immunological signatures in females but not males. Furthermore, females have a much wider chromosomal distribution of their sexually dimorphic genes compared to males” [18]. As a whole, these findings point to the complex underlying genetics and epigenetics that influence GBM pathophysiology that, if better understood, holds great potential in developing more effective treatment strategies.

Our findings confirm that females have a higher prevalence of MGMT promoter methylation compared to males. MGMT promoter methylation is seen in 52–56% of females compared to 37.4–47% in males [4,10,11]. These clinical observations are consistent with pre-clinical drug studies, which demonstrated that female GBM cells were more sensitive to temozolomide [20]. Although not significant in PFS comparisons, our data suggests a greater PFS and OS benefit conferred in females by MGMT promoter methylation. This PFS outcome may be due to inconsistencies in determining progression versus pseudo-progression in females following radiation and temozolomide. Although multivariate analysis diminishes the prognostic significance of sex-associated MGMT promoter methylation, the trend towards improved PFS and OS in females is intriguing for its potential clinical relevancy. We are currently looking to validate and expand upon this study in a larger cohort. The distinctions between male and female GBM need to be further investigated. Among the next steps in identifying these distinctions will be re-evaluating whether sex-dependent clinical cutoff values exist. The less distinct stratification of PFS and OS by MGMT promoter methylation among males raises the possibility that the cutoff for defining methylation status in males could be better optimized. Additional steps might also be taken to further investigate potential sexual dimorphism in recurrent GBM and other tumor types, which have thus far shown little clinical significance [27,28].

This work has certain limitations, primarily being the retrospective nature of the investigation. Additionally, not every single predictor of survival was adjusted for in the multivariable model. However, this sex-associated difference in MGMT methylation is among the emerging sex differences in GBM, both in the context of incidence and survival, with males having a 1.58-fold higher incidence and poorer prognosis [20]. Additionally, given that the study was conducted on patients enrolled between 2012 to 2018, when the WHO 2021 classification of CNS tumors had not yet been implemented, therefore, an integrated histo-molecular classification approach was not utilized for diagnosis here.

Overall, these methylation differences provide a foundation for a more detailed assessment of epigenetic changes that are likely to be as apparent as sex differences in the genetics between male and female GBM [16,20] that underlie differences in imaging and survival [9]. Expansion of these sex differences assessments to other aspects relevant to GBM growth, progression, and therapeutic response, including alterations in metabolism and immune response, may provide an opportunity for the development of more sex-specific biomarkers and the identification of new pathways amenable for targeting [18,29,30].

## 5. Conclusions

Findings from this study validate those from other groups regarding MGMT promoter methylation being more common in females and promoter methylation having a larger positive impact on survival in females compared to males. Females had higher mean and individual CpG site methylation and received a greater PFS and OS benefit by MGMT methylation, which was not seen in males despite equal degrees of CpG methylation. These findings underscore the potential benefit of integrating sex-specific differences into clinical decision-making and provide a foundation for additional studies investigating both sex-specific mechanisms driving GBM progression and therapeutic approaches that could be integrated into personalized medicine strategies.

## Figures and Tables

**Table 1:** Baseline patient characteristics at time of surgery, overall and stratified by sex (*n* = 464).

Characteristic	Total	Female	Male
	(*n*= 464)	(*n* = 170)	(*n* = 294)
**Mean age (SD)**	63.4 ± 12.0	64.0 ± 11	63.0 ± 12.4
**Patients aged** **≥ 65, *n* (%)**	213 (45.9)	82 (48.2)	131 (44.6)
**Race, White** *, *n* (%)	414 (91.4)	150 (90.4)	264 (92.0)
**MGMT methylated, *n* (%)**	197 (42.5)	87 (51.2)	110 (37.4)
**Surgery type, *n* (%)**			
Biopsy	331 (71.3)	119 (70.0)	212 (72.1)
Resection	133 (28.7)	51 (30.0)	82 (27.9)
**KPS before surgery, *n* (%)** **			
≤80	80 (17.2)	33 (19.4)	47 (16.0)
90–100	96 (20.7)	28 (16.5)	68 (23.1)
Unknown	288 (62.1)	109 (64.1)	179 (60.9)
**KPS after surgery, *n* (%)** *			
<80	204 (48.0)	82 (51.6)	122 (45.9)
80	111 (26.1)	40 (25.2)	71 (26.7)
90–100	110 (25.9)	37 (23.3)	73 (27.4)
**IDH mutation status, *n* (%)**			
No	370 (79.7)	137 (80.6)	233 (79.3)
Yes	22 (4.7)	11 (6.5)	11 (3.7)
Unknown	72 (15.5)	22 (12.9)	50 (17.0)
**EGFR amplification, *n* (%)**			
No	235 (50.6)	86 (50.6)	149 (50.7)
Yes	161 (34.7)	66 (38.8)	95 (32.3)
Unknown	68 (14.7)	18 (10.6)	50 (17.0)
**Ki67, ≤40%**	269 (59.6)	180 (62.7)	89 (54.3)
**Steroid use** *, *n* (%)			
No	9 (2.2)	4 (2.6)	5 (1.9)
Yes	408 (97.8)	150 (97.4)	258 (98.1)

* Data not available for all subjects: White race (*n* = 11), steroid use (*n* = 47), KPS after surgery (*n* = 39), Ki67 (*n* = 13). ** Significant missing data for KPS before surgery due to logistical reasons, hence row of “unknown” status presented separately. Values presented as mean ± SD or *n* (column %).

**Table 2:** Patient characteristics overall and stratified by MGMT promoter methylation status.

Factor	Total (*n* = 464)	MGMT Un-Methylated (*n* = 267)	MGMT Methylated (*n* = 197)	*p*-Value
**Mean age**	63.4 ± 12.0	62.0 ± 11.7	65.2 ± 12.0	**0.004 ^a^**
**Patients aged ≥ 65**	213 (45.9)	106 (39.7)	107 (54.3)	**0.002 ^c^**
**Sex, female**	170 (36.6)	83 (31.1)	87 (44.2)	**0.004 ^c^**
Race, White *	414 (91.4)	243 (93.1)	171 (89.1)	0.13 ^c^
**Complete resection surgery**	133 (28.7)	72 (27.0)	61 (31.0)	0.35
Steroid use *	408 (97.8)	232 (97.1)	176 (98.9)	0.21 ^c^
Ki67 ≤ 40 *	0.007	169 (65.0)	100 (52.4)	**0.007 ^c^**
**IDH mutation status**				**0.002 ^c^**
No	370 (79.7)	211 (79.0)	159 (80.7)	
Yes	22 (4.7)	6 (2.2)	16 (8.1)	
Unknown	72 (15.5)	50 (18.7)	22 (11.2)	
**EGFR amplification**				0.25 ^c^
No	235 (50.6)	129 (48.3)	106 (53.8)	
Yes	161 (34.7)	93 (34.8)	68 (34.5)	
Unknown	68 (14.7)	45 (16.9)	23 (11.7)	

* Data not available for all subjects: race (*n* = 11), steroid use (*n* = 47), Ki67 (*n* = 13). Values presented as mean ± SD or N (column %). *p*-values: a = ANOVA, c = Pearson’s chi-square test.

**Table 3:** Univariable and multivariable Cox proportional hazards analysis of factors associated with overall survival (*n* = 464).

Variable	*n*	Events	Median OS, Months	2-Year OS, % (95% CI)	Univariate HR (95% CI)	Univariate Wald *p*-Value	Cox Multivariable HR (95% CI)	Multivariable Wald *p*-Value
**Sex and MGMT group**								
Un-methylated/female	83	68 (82%)	9.5	11.1 (2.9, 19.3)	1.98 (1.39, 2.82)	<0.001	2.07 (1.45, 2.95)	<0.0001
Methylated/female	87	59 (68%)	18.7	36.8 (25.3, 48.3)	1		1	
Un-methylated/male	184	151 (82%)	11.3	12.2 (6.8, 17.6)	1.72 (1.27, 2.33)	<0.001	2.14 (1.57, 2.93)	<0.0001
Methylated/male	110	78 (71%)	12.4	24.3 (14.4, 34.1)	1.45 (1.03, 2.04)	0.032	1.42 (1.01, 1.99)	0.04
Un-methylated/male vs. methylated/male	1.19 (0.90, 1.56)		0.22		1.51 (1.14, 2.00)		0.004	
Un-methylated/female vs. un-methylated/male	1.15 (0.86, 1.53)		0.34		1.04 (0.77, 1.39)		0.81	
**Age at surgery**								
<65	251	180 (72%)	15.0	26.1 (19.8, 32.4)	1		1	
≥65	213	176 (83%)	7.8	11.2 (6.2, 16.3)	1.85 (1.50, 2.29)	<0.001	2.25 (1.8, 2.81)	<0.0001
**Race**								
Other race	39	31 (79%)	11.5	14.4 (1.6, 27.2)	1			
White	414	318 (77%)	12.2	19.7 (15.3, 24.2)	0.93 (0.64, 1.34)	0.68		
**Surgery**								
Incomplete resection (partial/biopsy)	331	270 (82%)	8.5	14.3 (10.0, 18.6)	1.90 (1.49, 2.43)	<0.001	2.10 (1.64, 2.69)	<0.0001
Complete resection	133	86 (65%)	17.1	32.4 (22.7, 42.2)	1		1	
**Ki67 Proliferation index**								
≤40%	269	206 (77%)	10.3	15.1 (9.9, 20.2)	1.18 (0.95, 1.46)	0.13		
>40%	182	141 (77%)	12.8	23.6 (16.6, 30.5)				
**Steroid use**								
0: No	9	8 (89%)	15.3	25.9 (0.0, 56.6)	1			
1: Yes	408	312 (76%)	12.8	20.6 (16.1, 25.2)	1.17 (0.58, 2.37)	0.65		

**Table 4:** Effect of MGMT methylation on OS between CpG-matched male and female.

CpG/Sex Group	*n*	Mean CpG, Median (IQR)	Death, *n* (%)	Median (mo)	1-Year OS% (95% CI)	Log-Rank *p*-Value	Cox Univariate Hazard Ratio (95% CI)	Cox Univariate Wald *p*-Value
Before matching *						0.002		
cpg < 12/female	52	3.0 (2.0, 3.0)	42 (81%)	9.5	35.9 (21.7, 50.1)		2.36 (1.47, 3.78)	<0.001
cpg ≥ 12/female	52	41.5 (23.5, 59.0)	31 (60%)	18.9	68.0 (54.9, 81.0)		--	
cpg < 12/male **	132	2.0 (2.0, 3.0)	105 (80%)	11.0	45.3 (36.1, 54.4)		1.95 (1.30, 2.93)	0.001
cpg ≥ 12/male	68	37.5 (22.5, 50.5)	45 (66%)	12.4	53.3 (40.8, 65.7)		1.603 (1.012, 2.540)	0.04
After matching *						0.02		
cpg < 12/female	50	2.5 (2.0, 3.0)	40 (80%)	10.0	37.4 (22.8, 52.0)		2.54 (1.35, 4.77)	0.004
cpg ≥ 12/female	26	35.0 (18.0, 46.0)	13 (50%)	18.7	78.4 (61.3, 95.5)		1	
cpg < 12/male **	50	2.5 (2.0, 3.0)	38 (76%)	13.6	56.7 (41.8, 71.6)		1.78 (0.94, 3.37)	0.08
cpg ≥ 12/male	26	35.0 (18.0, 46.0)	19 (73%)	13.0	56.0 (36.4,75.6)		1.64 (0.81,3.33)	0.17

* *p* (CpG-sex interaction) 0.03 before match, 0.048 after match; ** *p* (cpg < 12/male vs. cpg ≥ 12/male) 0.27 before match, 0.76 after match.

## Data Availability

Data that were used for the results, tables, and figures, will be made available to qualified investigators upon reasonable request to the corresponding author (manmeeta@baptisthealth.net) after approval by all required regulatory authorities.
